# The effect of pregnancy on orthodontic tooth movement in rats

**DOI:** 10.4317/medoral.18465

**Published:** 2012-12-10

**Authors:** Kavoos Ghajar, Pooya Olyaee, Behnam Mirzakouchaki, Loghman Ghahremani, Alireza Garjani, Esmaeil Dadgar, Sahel Marjani

**Affiliations:** 1 DDS, Resident of Endodontics, Hamedan University of medical sciences, Hamedan, Iran; 2 DDS, MSc, Assistant Professor, Department of orthodontics, Faculty of dentistry, Urmia University of Medical Sciences, Urmia, Iran; 3 DDS, MSc, Associate Professor, Department of orthodontics, Faculty of dentistry, Tabriz University of Medical Sciences, Tabriz, Iran; 4 DDS, MSc, Assistant Professor, Department of Prosthodontics, Faculty of dentistry, Urmia University of Medical Sciences, Urmia, Iran; 5 PhD, Professor, Faculty of Pharmacy, Tabriz University of Medical Sciences; 6 DDS, Private practice, Tabriz, Iran; 7 DDS, Private practice, Hamedan, Iran

## Abstract

Introduction: The purpose of this study was to investigate the effect of pregnancy on orthodontic tooth movement in Wistar rats. Material and Methods: Forty eight female three-month old Wistar rats with an average weight of 250±25 gr were selected and randomly divided into two experimental (pregnant) and control groups (non-pregnant). Maxillary central incisors were tipped distally by insertion of springs exerting 30g force. Two, seven and fourteen days after spring insertion animals were sacrificed. Then the mesioincisal distance between maxillary incisors were measured. Subsequently, histological sections were prepared to count osteoclasts under a light microscope. The data on the extent of orthodontic tooth movement, and the number of osteoclasts were analyzed by independent sample t-test. Results: The results indicated that 2,7 and 14 days after force application there was no significant difference in orthodontic tooth movement between experimental and control groups (p>0.05). The number of osteoclasts were significantly lower in the experimental group 7 and 14 days after spring insertion (p<0.05). Conclusion: Pregnancy may decrease the amount of tooth movement in the linear phase but it is not statistically significant. The number of osteoclasts is significantly decreased during pregnancy.

** Key words:**Pregnancy, rat, orthodontic tooth movement, osteoclast.

## Introduction

Orthodontically induced tooth movement is the normal result of applying a mechanical force to a tooth. In the general view of orthodontic tooth movement, bone formation is associated with the tension side and resorption with the compression side. Three fundamental biologic responses are attributed to orthodontic loads: bone formation, bone resorption, and iatrogenic external root resorption (1).

Female patients may become pregnant before or during the treatment, and in this prolonged process, the level of sex hormones will reach to higher levels than non- pregnant patients. The previous studies indicate that these hormones have physiologic effects on bone metabolism, skeletal formation, and maintaining bone equilibrium in adults (2).

According to relation between sex hormones and tooth movement, pregnancy might influence the amount of tooth movement, duration of orthodontic treatment, applied force quantity and the side effects of tooth movement(3).

The purpose of the present study was to investigate the effect of pregnancy on orthodontic tooth movement in a rat model.

## Material and Methods

A total of forty eight 3-month-old female Wistar rats, with an average weight of 250±25gr, were used in this study. Animals were examined in two groups; experimental (n=24) and control (n=24).Each group was divided into three subgroups of 8 each.

The rats in the experimental group were pregnant, and controls were non-pregnant. All the rats had intact maxillary central incisors, and had no oral lesions. Determination of pregnancy was with clinical examination and abdomen expansion, subsequently confirmed by dissection and observation of the fetuses in womb after sacrificing.

Animals were acclimatized for 7 days in plastic cages (two per cage) with a standard 12-hour light/dark cycle. The temperature was maintained at 25º C and humidity at 50%. Animals were fed with a diet of finely ground laboratory food ad libitum, and had access to drinking water. The rats were anesthetized with an intraperitonal injection of ketamine (50 mg/kg) before spring insertion.

Orthodontic force was administered to all groups to distalize maxillary central incisors. Springs were bent from 0.35-mm stainless steel wire (Dentaurum, Germany) with a length of 8 millimeters, modified in the light of the previous studies by Mirzakouchaki and Firoozi (4).The springs were placed on a grid and activated on a single arm with pliers. The force was measured with a gauge (ETM, America). Springs were attached by means of the bands (Dentaurum, Germany), and a 30 g reciprocal force was applied to the teeth. The springs were not reactivated during the experiment.

The distance between the mesial corners of the maxillary incisors was measured by each author before appliance insertion with a digital caliper with accuracy of 0.01 mm. The appliance was placed on maxillary central incisors using an orthodontic band seater, and glass ionomer cement (Bandtite, America). The bands had a 3 mm distance with the incisal edge of the teeth, incisocervically. A thin spacer (0.05 mm) was placed between the central incisors before cement setting to prevent adhesion of the centrals to each other.

Two, seven and fourteen days after appliance insertion, 8 rats from each group (experimental and control) were randomly sacrificed by overdose of anesthetic drug. The distance between the mesial corners of maxillary central incisors was measured.

After sacrificing the rats, their premaxillae were dissected and placed in 10% formalin for four days. After fixation, the springs were removed, and the premaxillae were decalcified with 5% nitric acid for two days. The decalcified premaxillae were fixed again in the same manner for another three days. The sample was then dehydrated in a graded series of ethanol and embedded in paraffin (passage process). Then processes of clearing and impregnation were carried out. The paraffin blocks were sectioned serially using a microtome (Leitz, Germany), at 5 micrometer intervals in the frontal plane.

Five serial sections, each 5 µm thick were obtained 400 µm away from the alveolar crest. The sections, including maxillary central incisors (left and right), their alveolar bone and nasal septum were obtained perpendicular to the roots of the teeth. The sections were mounted on glass microscope slides and stained with hematoxylin and eosin. The osteoclasts were counted in a 1mm2 surface under a light microscope. Osteoclasts were identified as cells situated at the bone surface and containing more than three nuclei, a large amount of cytoplasm, and location in a resorption cavity. The Institutional Ethics Committee of Tabriz Medical University granted ethical clearance and the study followed the Helsinki guidelines prescribed for animal experimentation.

The data were analyzed by SPSS 14 software (SPSS for Windows, SPSS, Chicago, USA).Descriptive statistics are given as means±SEM. Smirnov-Kolmogrov test was employed to investigate normal distribution. Independent sample t-test was used to compare the amount of tooth movement, and osteoclast number between experimental and control group (p<0.05 is considered significant). One- way ANOVA, and Tukey test for post hoc was used to compare the amount of tooth movement and osteoclast number between 2nd, 7th and 14th day after appliance therapy in each group.

Results

After employing Kolmogrov-Smirnov test the data showed normal distribution. All of the data are expressed as means±SEM.

1- The distance between mesioincisal edges of maxillary central incisors (amount of tooth movement) after two days of appliance therapy was 0.77±0.04 in the control group and 0.56±0.10 in the experimental group. Independent sample t-test showed that there was no significant difference (p=0.99) in the amount of tooth movement between control and experimental group after two days.

2- The amount of tooth movement after seven days of appliance therapy was 1.00±0.17 in the control group and 0.78±0.12 in the experimental group. There was no significant (p=0.31) difference in the amount of tooth movement between control and experimental group after seven days.

3- The amount of tooth movement after fourteen days of appliance therapy was 1.64±0.17 in the control group and 1.26±0.11 in the experimental group. There was no significant difference in the amount of tooth movement between control and experimental group after fourteen days (p=0.95) ( Table 1).

**Table 1:**
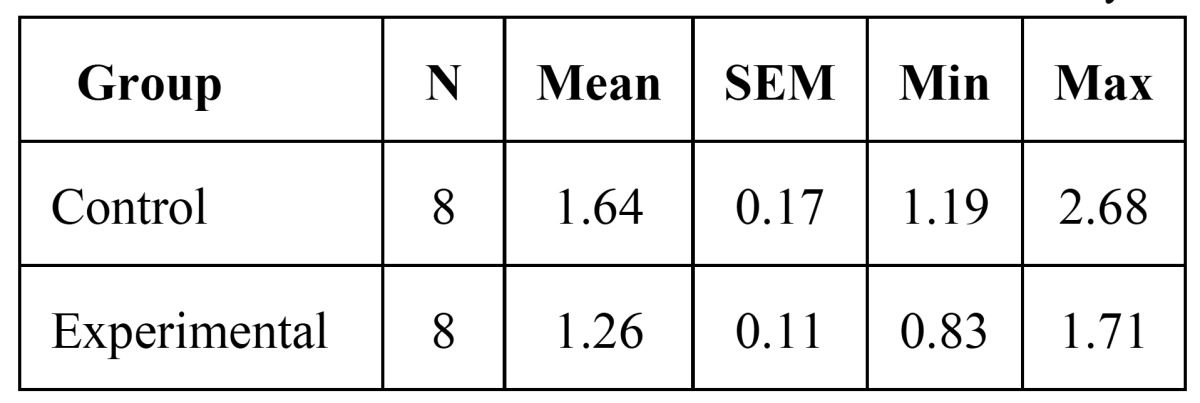
The amount of tooth movement after fourteen days.

4- The amount of tooth movement after two, seven and fourteen days of appliance therapy was 0.77±0.04, and 1.00±0.17 and 1.64±0.17 respectively in the control group. ANOVA test revealed that there was a significant difference in the mean amount of tooth movement in the control group (F(2,21)=9.53, p=0.001). Tukey (post hoc) test showed that there is a significant difference in the amount of tooth movement between days two and fourteen(p=0.001), and days seven and fourteen (p=0.015).

5- The amount of tooth movement after two, seven and fourteen days of appliance therapy was 0.56±0.10, and 0.78±0.12and 1.26±0.11 respectively in the experimental group. ANOVA test revealed that there was significant difference in the mean amount of tooth movement in the experimental group (p=0.001, F(2,21)=9.68). Tukey (post hoc) test showed that there is a significant difference in the amount of tooth movement between days two and fourteen (p=0.001), and days seven and fourteen (p=0.02).

6- The mean number of osteoclasts per square millimeter of microscopic field on the movement side (osteoclast number) two days after appliance therapy was 1.37±0.15 in the control group and 1.00±0.08 in the experimental group .Independent sample t-test revealed that there was no significant difference between control and experimental group (p=0.06).

7- Osteoclast number seven days after appliance therapy was 2.69 ±0.41 in the control group and 1.67 ±0.10 in the experimental group. There was a significant difference between control and experimental group (p=0.04) ( Table 2).

**Table 2:**
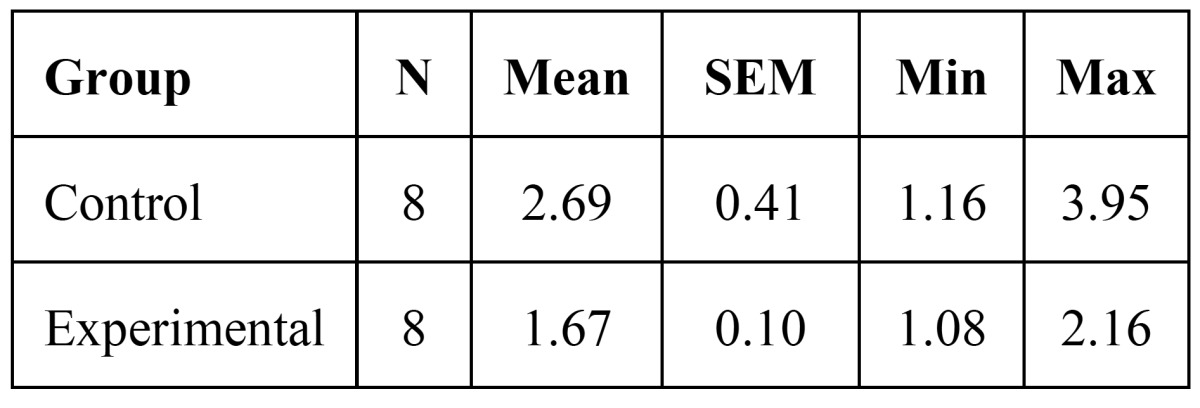
Osteoclast number after seven days.

8- Osteoclast number fourteen days after appliance therapy was 3.15 ±0.15 in the control group and 1.95 ±0.16 in the experi-mental group . There was a significant difference between control and experimental group (p<0.0005)  Table 3).

**Table 3:**
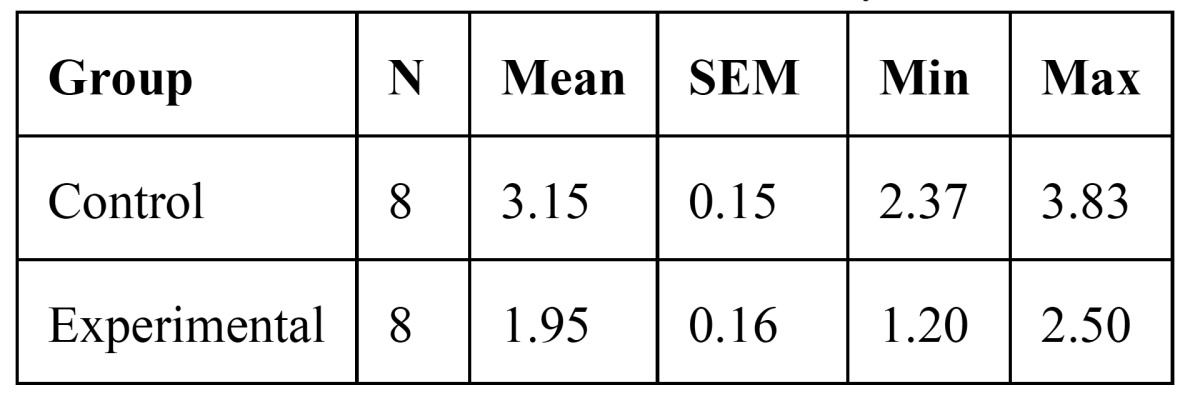
Osteoclast number after fourteen days.

9- Osteoclast number after two, seven and fourteen days of appliance therapy were 1.37±0.15, and 2.69±0.41 and 3.15±0.15 respectively in the control group. ANOVA test revealed that there was a significant difference in the mean amount of tooth movement in the control group (F(2,21)=11.43, p<0.0005). Tukey (post hoc) test showed that there is a significant difference in the amount of tooth movement between days two and seven (p=0.007), and days two and fourteen (p<0.0005).

10- Osteoclast number after two, seven and fourteen days of appliance therapy was 1.00±0.08, and 1.67±0.10 and 1.95±0.16 respectively in the experimental group. ANOVA test revealed that there was a significant difference in the mean amount of tooth movement in the control group (F(2,21)=14.81, p<0.0005). Tukey (post hoc) test showed that there is a significant difference in the amount of tooth movement between days two and seven (p=0.002), and days two and fourteen (p<0.0005).

## Discussion

In the present study, pregnancy caused decreased tooth movement, which was not statistically significant. Pregnancy also resulted in a significantly decreased number of osteoclasts at the movement side seven, and fourteen days after force application.

Different studies were done about pregnancy, bone turn over, osteoblasts activity and sex hormones before, but the relationship between bone turn over and pregnancy, and orthodontic tooth movement has not been clearly defined (3,5-7).

Hessling has shown increased orthodontic tooth movement during pregnancy (3). While Syed revealed that estrogen inhibits bone remodeling by concurrently suppressing osteoblastogenesis and osteoclastogenesis from marrow precursors (8).

Our findings are in contrast with Hessling, they reported increased osteoclasts numbers during pregnancy (3) and also in contrast with the studies that indicated sex hormones specially estrogen slow down orthodontic tooth movement (9), this may be due to the difference in clinical conditions of the latter studies and the fact that in pregnancy estrogen level is not the only variable.

The time-course of tooth movement is known to have the following three characteristic phases in rats: instantaneous tooth movement as the first phase, delayed tooth movements the second one, and a linear increment of tooth movement as the third one (10).

In the present study, instantaneous tooth movement phase was observed during the first two days and could be due to viscoelastic properties of the periodontal tissues in the first stage (11). No significant difference was noticed in the amount of tooth movement two days after force application between control and experimental groups; this may be because the amount of tooth movement during the instantaneous phase is due to viscoelastic properties of the periodontal ligament. The delayed tooth movement phase was observed between the third and seventh days after force application. There was also no significant difference between the amounts of tooth movement seven days after force application. Thereafter, a linear increment of tooth movement was observed in our study, and the alveolar bone remodeled with a balanced sequence of bone formation and resorption in this phase (12). There was no significant effect of pregnancy on the rate of tooth movement during the instantaneous, delayed and linear increment of tooth movement phases. This finding suggests that tooth movement in each phase was decreased but metabolic changes resulted from pregnancy were not enough to cause this significant. However we can expect decrease of orthodontic tooth movement in clinical practice. This process seems to be time dependent. Future studies are needed to investigate how long does it take for pregnancy to reduce alveolar bone turnover, and consequently tooth movement in human beings.

The present appliance was designed to produce a continuous horizontal force, and caused a tipping movement of the teeth. Once the appliance had been adjusted to produce 30 gram of force before installation, no activation was necessary during experimental tooth movement, and no deformity of the appliance was noted in any of the experimental rats at its removal. Tipping movement of the teeth results in pressure and tension at the periodontal ligament. It has been confirmed that the periodontal ligament is stretched and compressed at tension and pressure sites, respectively, 24 hours after force application. However, when the tooth movement shows a linear increment the width of the PDL is almost constant. Moreover, similar activation of both bone formation, and resorption at pressure sites is observed and confirmed by histomorphometry. It has been suggested that an increased rate of experimental tooth movement could activate bone remodeling in a coupled sequence of formation, and resorption (10).

In the present study osteoclast number at the movement side was selected as a quantitative index of alveolar bone remodeling. Histomorphometric analysis showed a significantly decreased number of osteoclasts at the movement side, seven, and fourteen days after force application.

Osteoclasts are primarily observed two days after force application. We noticed lower number of osteoclasts two days after appliance insertion in the experimental group although it was not significant. This decrease in osteoclast number may be due to gradual increase of estrogen and progesterone at early phases of pregnancy. It has been suggested that maximum osteoclast recruitment happens five to seven days after force application (13). In the present study osteoclast recruitment was significantly lower seven days after force application and also during the linear phase after fourteen days. These findings show a reduced rate of bone remodeling at the movement side, during pregnancy because of high level of sex hormones.

Estrogen inhibits bone remodeling by concurrently suppressing osteoblastogenesis and osteoclastogenesis from marrow precursors. Estrogen inhibits bone resorption via effects on the RANKL/RANK/osteoprotegerin system, as well as by reducing the production of a number of pro-re sorptive cytokines (such as IL-1, IL-6), along with direct effects on osteoclast activity and lifespan (8). In a review by Thijssen (14), it has been concluded that there are no indications that the various progestins, used in clinical practice, have either a bone-protective or an estrogen antagonistic activity. Progestins do not add or subtract much of the protective action of estrogens on the bones. During experimental tooth movement in the present study, pregnancy resulted in a significant decrease in the number of osteoclasts. In our opinion this effect is mostly because of the increased estrogen and progesterone level.

## Conclusion

In the present study it was observed that pregnancy in Wistar rats a little bit reduced orthodontic tooth movement, although it was not statistically significant, but osteoclasts number was significantly decreased at the movement side. Therefore, female patients should be informed that pregnancy may reduce their orthodontic tooth movement and subsequently increase treatment period, but they should not be worried about get in trouble during pregnancy and orthodontic treatment, because duration of orthodontic treatment is often more than pregnancy period, furthermore results of our study indicated no significant decrease in clinical amount of tooth movement.
